# A historical journey of metabolite–protein interaction discovery: from data harmonization to AI-driven prediction

**DOI:** 10.1093/bib/bbag355

**Published:** 2026-07-03

**Authors:** Toby Lawrence, Ema Mocsonokyova, Adwait Mahesh Barde, Dezso Modos, Marc-Emmanuel Dumas, Tamas Korcsmaros, Lejla Gul

**Affiliations:** Department of Metabolism, Digestion and Reproduction, Imperial College London, Du Cane Rd, London W12 0NN, United Kingdom; Department of Metabolism, Digestion and Reproduction, Imperial College London, Du Cane Rd, London W12 0NN, United Kingdom; Department of Metabolism, Digestion and Reproduction, Imperial College London, Du Cane Rd, London W12 0NN, United Kingdom; Department of Metabolism, Digestion and Reproduction, Imperial College London, Du Cane Rd, London W12 0NN, United Kingdom; Department of Metabolism, Digestion and Reproduction, Imperial College London, Du Cane Rd, London W12 0NN, United Kingdom; Institut Pasteur de Lille, Lille University Hospital, University of Lille, Rue du Professeur Calmette, 59000 Lille, France; Department of Metabolism, Digestion and Reproduction, Imperial College London, Du Cane Rd, London W12 0NN, United Kingdom; NIHR Imperial BRC Organoid Facility, Imperial College London, Du Cane Rd, London W12 0NN, United Kingdom; Quadram Institute Bioscience, Norwich, Norfolk NR4 7UQ, United Kingdom; Department of Metabolism, Digestion and Reproduction, Imperial College London, Du Cane Rd, London W12 0NN, United Kingdom; Quadram Institute Bioscience, Norwich, Norfolk NR4 7UQ, United Kingdom

**Keywords:** metabolite–protein interactions, metabolomics, cheminformatics, multi-omics integration

## Abstract

Metabolites are life-sustaining small molecules produced by living organisms**.** They interact with proteins involved in metabolism, signalling, and gene regulation, called metabolite–protein interactions (MPIs). This review traces the history of MPI research, from curated resources and early cheminformatics to harmonized identifiers, proteome-scale structural models, and artificial intelligence–driven prediction, while highlighting persistent challenges that continue to limit mechanistic interpretation of metabolomics. Early small-molecule-protein interaction prediction tools (e.g. Molpat and Catalyst) and resources (e.g. ChEMBL and BindingDB) were typically biased towards drug-like molecules. As drug-centred research continued, a revolution in large-scale metabolomics enabled high-throughput profiling of metabolite levels across physiological and disease states. However, these advances also introduced major data integration challenges such as data fragmentation, unresolved metabolite identities, and limited physiological context. Subsequent metabolite-centric resources (e.g. HMDB) and high-throughput screens applied to MPI detection (e.g. thermal proteome profiling) have partially addressed this bias. Proteome-scale structure prediction (e.g. AlphaFold) has further incentivized research into the effects of metabolites on protein structure and function. Nevertheless, the complexity of the biological response also depends on, e.g. exposure, access, and target expression. Looking ahead, MPI research is likely to be shaped by structure-aware deep learning and the integration of MPIs with comprehensive single-cell multi-omics data and host–microbe modelling. These advances may turn metabolomic signals into causal, testable hypotheses, enabling robust systems-level MPI maps for identifying intervention points and designing new treatments. We propose a historically structured roadmap centred on standards-driven data integration and calibrated, structure-aware modelling to support mechanistic, systems-level MPI maps.

## Introduction

Metabolites, defined here as small molecules under 1500 Da, are crucial physiological regulators produced endogenously by host biosynthesis and exogenously from the diet and the microbiome [1]. There are myriad types of metabolite protein interactions (MPIs). Metabolites can act as substrates, products or allosteric regulators of enzymes, regulators of transcription factors, serve as intermediate signalling molecules and co-factors, and be moved across membranes by dedicated transport proteins at the cell and organelle level. Extracellularly, metabolites can bind membrane receptors such as G-protein coupled receptors to initiate signalling cascades. They may enter the cell via active transport, diffusion, or pinocytosis, and subsequently interact with intracellular proteins, which have a wide range of complex roles from homeostasis to replication [2].

In complex clinical conditions, such as inflammatory bowel disease, cardiometabolic disorders, and cancer, shifts in metabolite profiles are strongly associated with diseases [3, 4]. Over time, the field has moved from association-based analyses towards mechanistic mapping, yet we still lack causal links between altered metabolite abundance, their protein targets, and the downstream cellular phenotypes they induce [5]. This gap limits the interpretation of metabolomics, hinders biomarker translation, and constrains therapeutic discovery, because without mechanistic links it remains difficult to prioritize targets, predict context dependence, and identify actionable points of intervention.

Rather than systematically benchmarking all tools or ranking methods independently of their historical setting, this review follows MPI research through a set of recurring themes: metabolome and proteome coverage, mechanistic depth, physiological relevance, scalability, and their role in systems models (Table 1). This perspective allows the history of the field to be viewed not only as a sequence of technical advances, but also as a series of attempts to overcome persistent limitations. Earlier methods provided strong evidence for individual interactions but were difficult to scale [6]. High-throughput and multi-omics approaches later expanded discovery but often made mechanistic interpretation less direct [7, 8]. More recent AI- and structure-driven methods can now generate candidate MPIs at much larger scale, but they also shift the main challenge towards confidence calibration, fair benchmarking, and biological interpretation [9]. By returning to these themes across each period, we aim to preserve the historical structure of the review while giving readers a clearer framework for understanding how different MPI approaches can be compared, interpreted, and applied in practice.

**Table 1:** Practical comparison of MPI discovery approaches across recurring interpretive themes.

**Approach**	**Coverage/scalability**	**Mechanistic depth**	**Physiological relevance**	**Role in systems models**	**Best practical use**
**Single-target assays**	Low; selected MPIs	High; direct binding or activity	Variable; often simplified *in vitro* systems	Limited alone but high confidence	Confirm direct binding, affinity, or function
**Experimentally resolved structures**	Low; tractable complexes	Very high; binding sites, poses, and contacts	Moderate; static structures	Strong for mechanistic understanding	Define binding mechanisms for key MPIs
**Curated CPI/MPI resources**	Moderate; uneven coverage	Variable; depends on evidence type	Often limited metadata	Useful as prior knowledge	Retrieve known interactions and build starting networks
**Metabolic/transporter DBs**	High for reactions/transport	Moderate; pathway or substrate context	Moderate; adds compartment/access information	Strong feasibility filtering	Add pathway, compartment, and transport constraints
**Ligand-based target prediction**	Broad; training-data biased	Low-moderate; target suggestions only	Low unless context is added	Hypothesis generation	Rapidly nominate possible targets
**Docking/co-folding**	Moderate-high if structures are available	Moderate; suggests binding modes	Low-moderate; often lacks cellular context	Prioritization and mechanistic hypotheses	Assess structural plausibility
**Molecular dynamics**	Low; selected candidates	High; captures dynamics	Moderate; more realistic but still simplified	Mechanistic refinement	Refine high-priority dynamic or allosteric MPIs
**High-throughput target engagement assays**	Moderate-high, depending on assay design	Moderate; detects binding or conformational response	Assay-dependent; lysate, cell, or tissue context	Experimentally supported networks	Discover condition-specific candidate MPIs
**AI-based prediction**	Very high; broad screening	Moderate; target or structure hypotheses	Low unless biologically contextualized	Expands candidate MPI networks but requires filtering	Rank candidate MPIs for follow-up analysis

To select representative papers, databases, and tools for each period, we used a structured, review-led approach rather than a formal systematic search. We began with widely used foundational resources across cheminformatics, metabolomics, structural biology, and systems biology and then used narrative reviews from each era to identify widely used methods and trends. We then identified follow-on methods and MPI-specific extensions by following citations from key papers and by prioritizing highly cited, MPI-relevant resources. We prioritized resources that are public and actively maintained where possible, and those that introduced new capabilities or data types (e.g. harmonized identifiers, high-throughput MPI screening, proteome-scale structural coverage, or AI-based prediction). We omitted enzyme kinetics and drug or xenobiotic metabolism, which have been extensively reviewed in recent publications [10–13].

**Figure 1:**
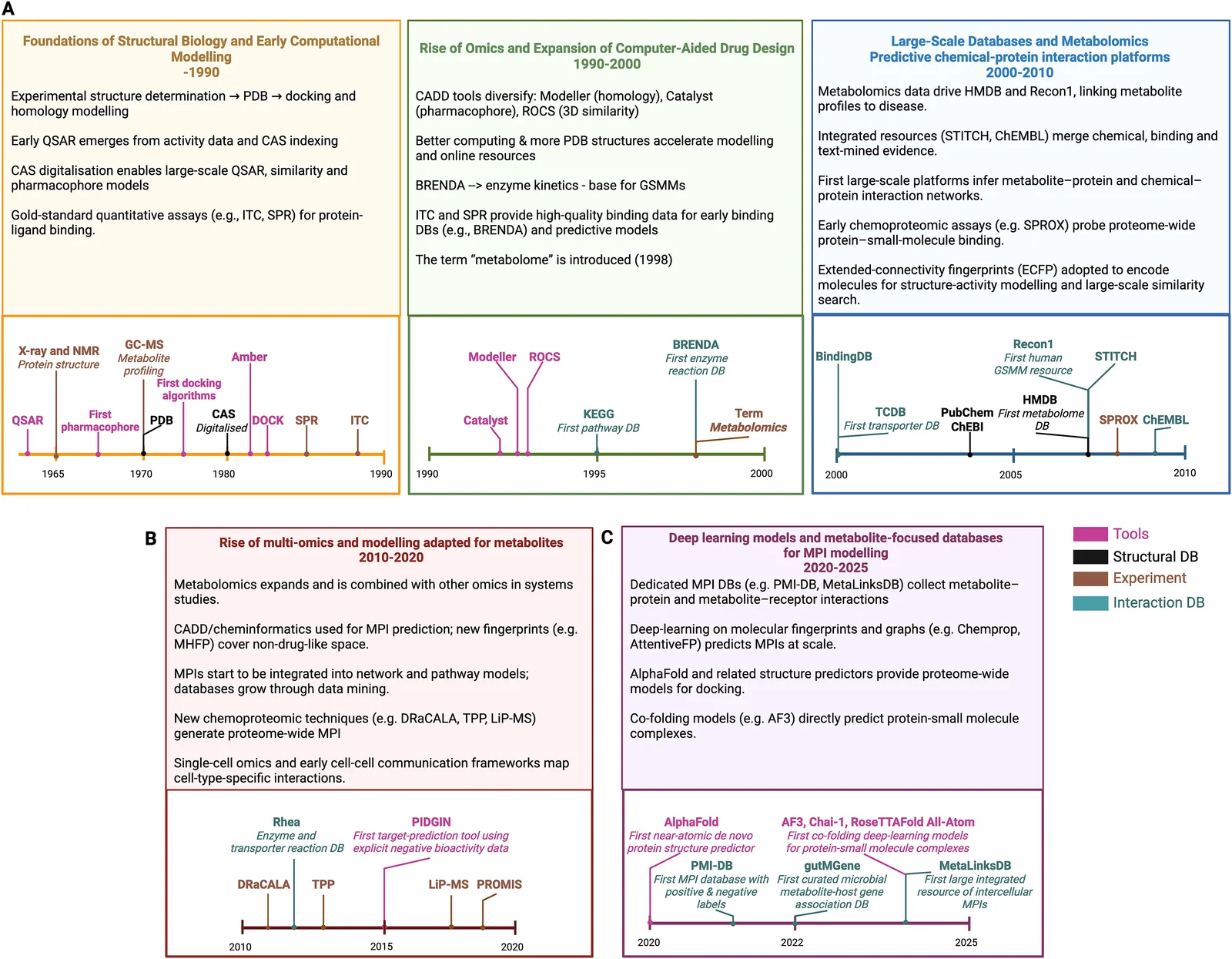
The history of metabolite-protein interaction research: (A) pre-2010; (B) 2010–20; and (C) 2020–5 era.

Using this historical perspective, we structure MPI discovery into three periods: a pre-2010 era dominated by single-target assays and drug-centred resources, a 2010–2020 phase characterized by high-throughput metabolomics and chemoproteomic methods, and a 2020–2025 phase in which larger datasets, proteome-scale structural prediction, and AI-driven models expanded candidate MPI mapping. We emphasize the development of improved experimental methods, available data resources, and data standards and how these can improve hypothesis generation, mechanistic understanding, and validation (Fig. 1). The following historical periods should therefore be seen not only as technological milestones, but also as responses to the methodological and biological challenges left unresolved by earlier approaches.

### Early metabolite–protein interaction discovery: bench assays, databases, and *in silico* models (pre-2010)

#### Single-target assays and structural biology of metabolite–protein interactions

Traditional methods for studying MPIs were often limited to single targets. These initial studies typically applied either high-resolution structural techniques or targeted functional and binding assays.

Some of the first methods providing atomic-resolution structures of ligand-bound protein complexes were NMR spectroscopy and X-ray crystallography [14, 15]. By the mid-1960s, co-crystallization of enzymes with small molecules was feasible, shown by lysozyme inhibited by the trisaccharide *N*-acetylglucosamine in 1965 [16]. These approaches resulted in high-confidence structural evidence for individual MPIs but only for a small subset of proteins and ligands.

In parallel, functional single-target studies used biochemical readouts such as enzyme activity and reporter assays, and were later complemented by biophysical techniques like Isothermal Titration Calorimetry (ITC) [17, 18] and Surface Plasmon Resonance (SPR) [19–21], to obtain affinities and kinetic parameters. Although ITC was developed decades earlier, it only became widely used for biomolecular interaction analysis after high-sensitivity instruments were developed in the late 1980s and early 1990s [17, 18], whereas SPR-based biosensors were first demonstrated in the early 1980s [19]. Single-target MPI studies used targeted biochemical and reporter assays to identify interactions such as short-chain fatty acids activating FFAR2/GPR43 and FFAR3/GPR41 [22, 23], butyrate inhibiting histone deacetylases [24], and bile acids engaging FXR [25, 26]. These interactions show how metabolites can rewire signalling and gene regulation. However, assay heterogeneity, limited sensitivity to weak/transient binding, sparse structural coverage, and poor physiological context were common limitations [6]. Much of the data was initially not collated into public resources and was confidential to pharmaceutical companies so overall coverage of MPIs remained limited and strongly drug-focused [27].

#### First wave of public resources

X-ray crystallography has historically provided some of the best curated and trusted scientific data. The Protein Data Bank (PDB), established in 1971, initially described seven protein structures (including oxygen and carbon monoxide bound to haemoglobin [28]) then expanded rapidly in the 1990s–2000s as more crystal and NMR structures were solved [29, 30]. By the end of 2025, there were 200 000 X-ray, 14 600 NMR, and 31 000 electron microscopic structures deposited in the PDB [31, 32].

Simultaneously, the Chemical Abstract Service (CAS) (maintained as hardcopy since 1907), provided a comprehensive database for chemical identifiers from 1980 [33]. By standardizing small molecule structures and nomenclature, CAS provided a foundation for later structure-based databases [34].

With the appearance of the internet, online databases appeared in the 1990s–2000s to centralize biological data which was previously fragmented [35]. Early chemical structure data resources appeared in this period, such as PubChem [36–38] and ChEBI (Chemical Entities of Biological Interest) [39, 40] which made chemical data on principally drug-like molecules more widely available. Concurrently, metabolite-centric structure databases, such as Kyoto Encyclopedia of Genes and Genomes (KEGG) compounds (1995) [41, 42], LIPIDMAPs (2003) [43], METLIN (2005) [44], and the Human Metabolome Database (HMDB—2007) [45, 46] were populated with available public metabolic data. Although early releases still covered only a subset of the metabolome, successive updates have greatly expanded coverage (for example, METLIN now contains >900,000 MS/MS spectra).

As the PDB has increased in size and diversity, there are now a large number of protein-ligand complexes, including metabolites. PDBbind (2004) has hyper-linked these structures to available binding affinities [47, 48]. These structural databases provided mechanistic insight into the experimentally determined binding sites of ligands. However, they still have relatively low coverage of MPIs. Databases such as the PDB and the associated small molecule database, the Cambridge Crystallographic Database, provide the fundamental data for modelling studies of protein–protein and protein-ligand (or metabolite) interactions [49].

For chemical-protein interactions (CPIs), databases expanded in waves. Early efforts such as BindingDB (conceptualized mid-1990s, public from 2000) [50, 51] focused on curated quantitative affinity data, describing how strongly specific small molecules bind particular targets. The Therapeutic Target Database (TTD, 2002 [52–54]) and DrugBank (2006 [55, 56]) then added a disease-centric view, emphasizing the clinically relevant proteins. From 2004, PubChem [36–38] increased the scale by aggregating heterogeneous screening data from many sources. Initially, these repositories were built by manually curating experimental information from medicinal chemistry and pharmacology experiments. Later releases included data from high-throughput screening and text-mining [37, 57] and led to large CPI platforms such as STITCH (2008) [57–59] and ChEMBL (2009) [60, 61]. ChEMBL was originally a commercial database, called StARlite developed by Inpharmatica Ltd, that was subsequently transferred to EMBL-EBI and made public [62]. Despite their size, these repositories remained primarily drug-centric, and endogenous metabolites represented only a small fraction of compounds in them due to the lack of annotated and characterized metabolite data [63] (Table 2).

**Table 2:** Databases describing metabolite–protein interactions.

**Resource**	**Scope**	**Limitations**
***Chemical–protein interaction resources***		
BindingDB [50, 51] (2000–6)	Quantitative binding data (e.g. affinities) from medical chemistry; large set of small molecule–protein interactions	Emphasis on drug-like ligands; poor metabolite coverage; no metabolite-specific filter
Therapeutic Target Database [52–54] (2002–6)	Resource on therapeutic targets with pharmacological/clinical data, including metabolites	Only metabolites linked to therapeutic targets; metabolite data are indirect; no metabolite-specific filter
PubChem [36–38] (2004–6)	Central hub aggregating bioactivity data and linking to metabolite DBs such as HMDB	Heterogeneous formats; no simple metabolite filter; strongly drug-discovery biased
DrugBank [55, 56] (2006–6)	Extensive information on drugs and targets, including receptors, enzymes, transporters, and carriers	Drug-centric; metabolites only when relevant to drug action/metabolism; no metabolite filter
STITCH [57–59] (2007–16)	Large CPI resource combining experimental data, database, and predicted interactions	No metabolite filter; predicted/text-mined links not systematically validated; last major update in 2016 (now in STRING)
ChEMBL [60, 61] (2009–25)	Chemogenomic DB with binding, functional, and ADMET data for bioactive, mainly drug-like molecules	Strong drug focus; literature bias to certain target families; only ‘natural product’ filter
Guide to Pharmacology [260] (2011–25)	Expert-curated ligand activity–target relationships, explicitly labelling metabolites	Smaller than other CPI sets; focuses on well-characterized pharmacology; limited MPI coverage
***Metabolite–enzyme interaction resources***		
KEGG [41, 42] (1995–2026)	Metabolic/pathway resource linking metabolites, enzymes, genes, and diseases via reactions	Emphasizes enzymatic reactions; few nonenzyme interactions; limited quantitative binding data
BRENDA [64–66] (1998–2025)	Curated EC-based enzyme database with substrates, products, inhibitors, and activators	Restricted to enzymes; no coverage of nonenzymatic proteins
MetaCyc [261] (1999–2025)	Experimentally validated metabolic pathways and catalysing enzymes, incl. substrates, inhibitors, and cofactors	Mainly qualitative; only partial kinetic data
Reactome [67–70] (2003–25)	Pathway DB for metabolic and signalling pathways, covering enzymes, transporters, and some allosteric regulation	For metabolites, information is largely qualitative with limited kinetic data; quantitative coverage is strongest for enzymatic steps
SABIO-RK [71–73] (2006–24)	Curated quantitative kinetic data for enzymatic reactions with inhibitors, activators, and cofactors	Enzyme-only; prioritizes kinetics over comprehensive binding coverage
***Transporter describing resources***		
Solute Carrier [76] (2004–19)	Qualitative data on solute carrier transporters and their transported substrates, including metabolites	Restricted to SLCs; lacks transport rates
TransportDB [77, 78] (2004–17)	Predicted membrane transport proteins and their substrates	Transporter-only; mainly predictive with limited experimental validation
TCDB [74, 75] (2005–21)	Classification/annotation of membrane transport proteins and their transported substrates	Transporters only; nonquantitative, function-focused
Rhea [142] (2012–26)	Curated catalytic and transport reactions with balanced stoichiometry and standardized ChEBI entities	Reaction-focused; omits receptor, allosteric, and TF binding; no quantitative affinity data
***Genome-Scale Metabolic Models***		
Recon1–3D [81, 140, 141] (2007–18)	Genome-scale human metabolic reconstructions for *in silico* analyses (e.g. flux balance analysis)	Limited to metabolism; little signalling or gene regulation; incomplete quantitative parameters
Human Metabolic Reaction [139] (2012–4)	Comparable to Recon but with a stronger emphasis on lipid metabolism	Like Recon, largely metabolic-centric; sparse signalling and regulatory coverage
Human-GEM [137] (2020)	Consensus reconstruction merging Recon and HMR, resolving duplications/inconsistencies	Still metabolism-focused; limited signalling and gene-regulatory content
***Structural binding databases***		
PDBbind [47, 48] (2004–25)	Links 3D biomolecular complexes in PDB to measured binding affinities	Biased to co-crystal/NMR complexes and drug-like ligands; sparse MPI coverage
***Metabolite-protein interaction databases***		
PMI-DB [187] (2021)	MPIs from large-scale MS-based assays for 23 small molecules across four species, including negatives	Limited metabolite set; no affinities or structural complexes
MetaLinksDB [186] (2024)	Integrates MPI-relevant data for signalling metabolites, with enzymatic links and biological context filters	Dependent on source DB quality; includes only one general CPI resource (STITCH)
MRCLinkdb [180] (2024)	Curated metabolite-receptor resources for cell–cell communication	Narrow interaction scope; smaller coverage than broader MPI resources

In parallel, enzyme-centric databases appeared to capture the metabolic context of MPIs. BRENDA began as a handbook in the 1990s then stored online in 1998 [64–66]. KEGG ENZYME (1995) and related KEGG resources linked enzymes, reactions, and metabolites, with the first paper in 2000 [41, 42]. Later other resources appeared, such as Reactome detailing comprehensive, manually curated information on human biological pathways extending from metabolism to signalling and other processes [67–70], while SABIO-RK [71–73] focused on detailed kinetic data. These databases focused on metabolic reactions, still primarily describing catalytic reactions rather than broader regulatory MPIs (Table 2).

Transporter-focused resources were then published to address access and compartmentalization, questions that classical CPI and metabolic databases could not fully answer. The Transporter Classification Database (TCDB) [74, 75], curated solute carrier (SLC) tables [76], and later TransportDB [77, 78] catalogued membrane proteins and their transported substrates, including metabolites and drugs. Genome-Scale Metabolic Models (GSMMs) also emerged at this time [79] beginning in 1999 with microorganisms such as *Haemophilus influenzae* [80] and later, in 2007, with human metabolism such as Recon1 and the Edinburgh Human Metabolic Network [81, 82]. GSMMs encode detailed reaction and transport information and are widely used for flux balance analysis, linking metabolite levels, enzyme activities, and transport processes [83]. However, they remain focused on metabolic stoichiometry and flux rather than capturing regulatory MPIs (Table 2).

### Early cheminformatic approaches for predicting metabolite–protein interactions

Because experimental MPI data were sparse, especially for nondrug metabolites, *in silico* models were advantageous to extend their applicability. Early work on computational MPI prediction centred on the transfer and adaptation of Computer-Aided Drug Design (CADD) methodologies and tend to fall into two major classes: ligand-based and structure-based approaches [84, 85].

#### Ligand-based target inference: the drug bias problem

Initially, CADD approaches were dominated by ligand-based methods, built on the molecular similarity principle that structurally similar molecules share similar biological activity [86]. Quantitative structure–activity relationships (QSARs), introduced by Hansch and Fujita in 1964 [87], encode ligands as numerical descriptors (e.g. hydrophobicity, polarity, size) and utilize statistical models to relate these features to biological activity (or physico-chemical properties such as logP) without knowledge of the target protein structure [88]. In the 1980s–90s, these concepts were scaled up, principally to accommodate high-throughput screening, and formalized into similarity searching. Similarity search infers targets finding the most structurally similar molecules in annotated databases and using their known targets or bioactivities to generate hypotheses for the query compound [89]. Molecules were encoded as fingerprints, compact, machine-readable bit strings of molecular structure, highlighting the presence of certain atoms, fragments, or features, and compared using similarity metrics such as the Tanimoto coefficient. Pharmacophore-based methods (e.g. Catalyst [90]) extended this concept to 3D, capturing spatial arrangement of key interaction features (e.g. hydrogen-bond donors/acceptors, hydrophobic regions, aromatic rings) [91, 92] (Table 3). These early ligand-based tools should therefore be understood as historically important extensions of drug-discovery logic rather than as metabolite-native MPI predictors. Because they were built on sparse, strongly drug-centred annotations, their limitations were not simply technical but conceptual. Many endogenous metabolites fell outside the chemical and biological assumptions on which these methods were trained.

**Table 3:** Ligand-based small molecule–protein interaction prediction tools.

**Method**	**Representative tools**	**Application**	**Metabolite-specific caveat**
3D similarity and pharmacophore	Catalyst [90]	Need for 3D analogues or a feature-based hypothesis (e.g. does this metabolite resemble known ligands in 3D?)	Flexible/polar metabolites can be poorly represented by a single conformer; protonation/tautomer and water-mediated binding can dominate; pharmacophores often reflect drug-like binding motifs
2D fingerprint similarity with statistical target inference	BioSEA [149]	Shared-target inference for metabolite-like compounds with close annotated molecules	Inherited drug-space bias and potentially missed metabolite-like binding
Bioactivity signature similarity	BASS [148]	Target deconvolution when compounds have rich assay panel readouts (profile-similarity driven)	Rare for endogenous metabolites due to sparse assay coverage; profiles can reflect indirect effects; requires comparable assay conditions
Drug-trained ligand-based target predictors	SuperPred [146]; OCEAN [147]	Rapid target prioritization for drug-/xenobiotic-/natural product–like compounds	Performance tied to overlap with drug-like training space; less reliable for small, highly polar/charged metabolites; treating more as prioritization than ground truth
Classical ML with explicit negatives	PIDGIN [150]	Interpretable target classification when curated noninteraction data are important for model training	Still constrained by training chemistry; metabolite domain shift persists unless retrained/fine-tuned on MPI-relevant labels and matched negatives
Deep learning on molecular graphs	Chemprop [193]; AttentiveFP [189]	MPI prediction when sufficient labelled data are available and nonlinear structure–activity patterns are likely to exceed fixed-descriptor models	Model architecture alone is not sufficient for robust metabolite-specific performance; outcomes depend on metabolite-relevant training data, harmonized labels, and rigorous benchmark design
Natural product/metabolite-like target inference	CTAPred [262]	Target prediction for natural product-, dietary-, and microbiome-related molecules beyond classic drug-like chemical space	Predictions weaken for metabolites outside well-covered natural product/drug-adjacent chemistry

The shift towards high-throughput screening has provided a large volume of data that enabled ligand-based modelling at scale [93]. Standardized molecular representations (e.g. canonical SMILES and InChI) enabled the computation of molecular descriptors such as fingerprints and physicochemical properties [94]. These descriptors serve as inputs for machine learning (ML) models trained to predict biological activity, thereby enabling large-scale *in silico* screening of chemical libraries. A number of these tools were incorporated into workflows that enable virtual screening on remote web-services [95]. However, there is a major caveat due to the drug-centric discovery: ligand-based models inherited a strong drug bias and covered only a small region of the metabolite chemical space.

#### Early structure-based modelling: fragmented data constraint

Structure-based CADD methods exploit the 3D structure of protein targets. In the pre-2010 era, experimentally determined structures, primarily from X-ray crystallography and NMR spectroscopy, with smaller contributions from neutron diffraction and electron crystallography, provided most of the 3D data used for structure-based CADD and were deposited in PDB [96]. Molecular docking is a structure-based computational technique that models the interaction between a ligand and a macromolecular target by sampling possible binding conformations within a defined binding site and evaluating them using a scoring function. Although early docking methods were developed in the 1980s, the approach became widely adopted as the number and quality of experimentally resolved protein structures increased, enabling systematic structure-based drug design [96, 97]. Programs like DOCK (1982) [98], AutoDock (1990) [99] and GOLD (1997) [100] generate and evaluate ligand poses within binding pockets, using empirical scoring functions to evaluate putative poses [101]. In the absence of experimentally resolved structures, homology modelling offers a structure-based strategy for approximating target protein architecture. Homologous proteins with known crystal or cryo-EM structures can be identified through sequence alignment tools such as BLAST [102], and these structures are subsequently used as templates to generate a three-dimensional model of the target. Model accuracy is strongly dependent on sequence similarity and conservation of structurally and functionally critical regions, especially the binding pocket. Representative implementations include MODELLER [103] and SWISS-MODEL [104]. These strategies greatly expanded the set of proteins for docking, but they also introduced additional uncertainties: higher sequence divergence or misplaced side chains in the model could alter the pocket from the true (present in the physiological environment of the cell, for example) defined shape [105]. To move beyond static docking approaches, molecular dynamics (MD) simulations have been used to model the time-dependent behaviour of protein-ligand interactions [106]. MD computes the atomistic motions of a system under a defined force field, allowing exploration of conformational flexibility, ligand stability within the binding pocket, and dynamic protein rearrangements. Such simulations can reveal transient conformations and occasionally expose cryptic binding sites that are not apparent in a single experimentally determined structure [107, 108] (Table 4).

**Table 4:** Structure-based small molecule–protein interaction prediction tools.

**Method**	**Representative tools**	**Application**	**Metabolite-specific caveat**
***Structure-based approaches***			
Molecular docking	DOCK [98]; AutoDock [99]; GOLD [100]	Used for pose hypotheses and geometric plausibility when screening a metabolite against a shortlisted set of targets; often a baseline step before refinement	Affinity ranking is unreliable; performance can drop for small/polar/charged/flexible metabolites; sensitive to protonation/tautomers; water/ions and metal coordination are often simplified; results depend strongly on pocket state
Homology modelling	MODELLER [103]; SWISS-MODEL [104]	Historically the main route to obtain 3D target structures for docking when experimental structures were missing; still used for template-controlled conformations, isoforms, and mutants	Limited when no suitable template exists; binding-site loops/disordered regions uncertain; may reflect template’s conformation (not the metabolite-bound state); often complemented (not fully replaced) by deep-learning structures
Molecular dynamics	GROMACS [263]	Used to refine a small number of high-priority MPIs, test pose stability, explore pocket opening/closing, solvent networks, and mechanism; sometimes used for free-energy style ranking on small sets	Computationally expensive; sampling limits (rare events/slow binding); metabolite parameterisation and charge/tautomer states can be challenging; results depend on force fields and setup choices
***Structural deep learning models***			
Protein structure prediction (single chain)	AlphaFold [199], RoseTTAFold [202], ESMFold [203], OmegaFold [204]	Provided near-proteome-wide protein structural coverage, enabling docking/pocket analysis for many targets that previously lacked structures	Does not directly model MPI complexes; predicted structures may miss ligand-induced conformations, allosteric states, or cofactor/ion-bound pocket geometries important for metabolite binding
Protein complex prediction	AlphaFold- Multimer [207]	Used to model protein assemblies so docking/analysis can account for quaternary structure and interfaces that shape pockets or access	Still not a ligand/metabolite complex model; interface errors can propagate to pocket geometry; ligand binding often depends on cofactors/ions or induced fit not captured in a single complex prediction
Biomolecular complex prediction	AlphaFold3 [191], RoseTTAFold All-Atom [209], Chai-1 [213], Boltz-2 [214], NeuralPLexer3 [215]	Emerging trend towards direct complex hypotheses (pose + contacts) for MPIs without explicit docking; useful for generating candidate-binding modes and prioritizing targets	Metabolite-relevant benchmarking is still limited; training/evaluation data are biased towards well-studied, often drug-like complexes; weak/transient binding and solvent/ion networks remain challenging; confidence scores may be poorly calibrated for metabolite-like ligands

Although these computational approaches had broader applicability, they have been most extensively developed and validated within the context of drug discovery. The reliability of docking predictions was strongly influenced by the accuracy of the receptor structure and binding-site definition. Furthermore, commonly used scoring functions exhibit reduced performance for small, polar, and conformationally flexible molecules, features characteristic of many endogenous metabolites [109]. Historically, computational resources were limited, and large-scale metabolomics technologies had not yet enabled systematic mapping of MPIs. Consequently, computational modelling efforts were primarily focused on drug-like compounds, and endogenous metabolites were typically investigated only when they exhibited properties compatible with established drug discovery workflows [6, 97]. For this reason, structure-based approaches from this period are best viewed as tools to suggest if an interaction is mechanistically possible rather than broadly applicable MPI discovery platforms. Their main value was to refine hypotheses for tractable drug targets, rather than accurately predicting MPIs across the whole metabolome.

### Appearance of high-throughput technologies

High throughput metabolite measurement provided a paradigm shift from drug-targeted interactions to broader MPIs [3]. In the early 1970s, Gas Chromatography Mass Spectrometry (GC–MS) work showed that metabolic profiles of human urine and tissue extracts could capture many metabolites simultaneously [110, 111]. These studies are often cited as the technical origin of metabolomics, although the terminology of metabolome, metabolomics, and metabonomics was only introduced later, during the late 1990s and early 2000s [112, 113].

From the late 1990s, advances in Liquid Chromatography Mass Spectrometry (LC–MS), GC–MS, and NMR [114], along with improved computational methods and databases [115], enabled systems-level metabolomics. These technologies connected metabolic shifts to physiology and disease supporting roles for specific metabolites in atherosclerosis, diabetes, inflammatory bowel disease, and other conditions [116, 117], and contributed to the recognition of altered metabolism as a hallmark of cancer [118]. Consequently, investigating MPIs to understand the downstream effects on the host gained importance [119].

### Challenges in the pre-2010 era

Till 2010, metabolomics increasingly revealed a central gap that metabolite-phenotype correlations rarely specify mechanism. Without explicit MPIs, it is difficult to explain how changes in metabolite concentration translate into altered signalling, enzyme regulation, or gene control in the relevant tissue or cell type. Early computational approaches quickly encountered practical problems. Historically, drug-focused bioactivity databases provided limited representation of endogenous MPIs, covering only a small subset of the chemically diverse metabolome. This restricted the applicability of ligand-based target prediction methods, which rely on annotated chemical-biological relationships within curated datasets. Structure-based approaches were likewise constrained by incomplete structural coverage of the proteome, especially the scarcity of ligand-bound conformations, and by scoring functions that have been more extensively calibrated and validated within drug-like chemical space than for small, polar, and flexible metabolites.

Taken together, the pre-2010 era established the biochemical and structural basis of MPI research, but its progress was concentrated on detailed studies of selected interactions rather than broad metabolome-wide coverage. The strongest evidence came from targeted assays and high-resolution structures, which offered high confidence for selected interactions yet provided limited metabolome- or proteome-wide coverage. In practical terms, this allowed the field to explain well-known cases of metabolite sensing and enzyme regulation, but not yet to interpret metabolomics data mechanistically at a broader scale. The central limitation of this period was therefore not the absence of mechanistic detail, but the inability to scale that detail beyond a small number of well-studied, largely drug-adjacent systems. Consequently, as high-throughput data became available, the field moved towards harmonized data integration and computational MPI inference, combining improved metabolite identification, reference resources, and proteome-scale structural information to prioritize causal, physiologically relevant MPIs for validation.

## Dawn of large-scale metabolomics: shifting from drug-centred to metabolite-centred systems studies (2010–20)

Building on the earlier technological advancements, the period from 2010 to 2020 moved from a drug-centred, single-target view of interactions towards a metabolite-centred, systems-level perspective. Around 2010, large-scale metabolomics and multi-omics began to report thousands of different metabolites together with associated genes and proteins, making MPIs valuable for connecting these signals. Systematic MPI mapping began to provide the missing link between correlation and causality by explaining how metabolites regulate protein function, signalling, and gene regulation [120]. Efforts focused on combining curated CPI and metabolic databases, computational prediction tools, and physiological context into metabolite-centred MPI maps that support mechanistic interpretation and hypothesis generation.

### Small molecules beyond drugs

Drugs and metabolites partially overlap as small molecules, but differ in origin, physicochemical properties, and biological roles [121]. Metabolites act within tightly regulated metabolic and signalling pathways, often through weak, transient, and context-dependent interactions [122]. In contrast, drugs are typically synthetic molecules designed to reach and persist at therapeutic targets, with medical chemistry optimizing both binding and ADME (absorption, distribution, metabolism, and excretion) properties [123]. On average, drugs are larger, more hydrophobic, and contain more ring structures than typical metabolites, which are smaller and more polar [124].

Drug-likeness historically incorporated Lipinski’s Rule of Five (Ro5), derived from successful oral drugs to enrich compounds with a higher probability of passive intestinal absorption. The Ro5 is a pragmatic filter for permeability-driven oral exposure, not a predictor of target engagement or intrinsic biological activity, and it does not explicitly represent ionization/microstate distributions or transporter-mediated processes. Neither do one or two rule violations doom a candidate, nor does full compliance guarantee bioactivity or developability. Although the field has moved beyond a strict Ro5 mindset, its influence still shapes screening collections, deposited actives, and many marketed drugs, thereby biasing what is synthesized, tested, and published [125].

**Figure 2:**
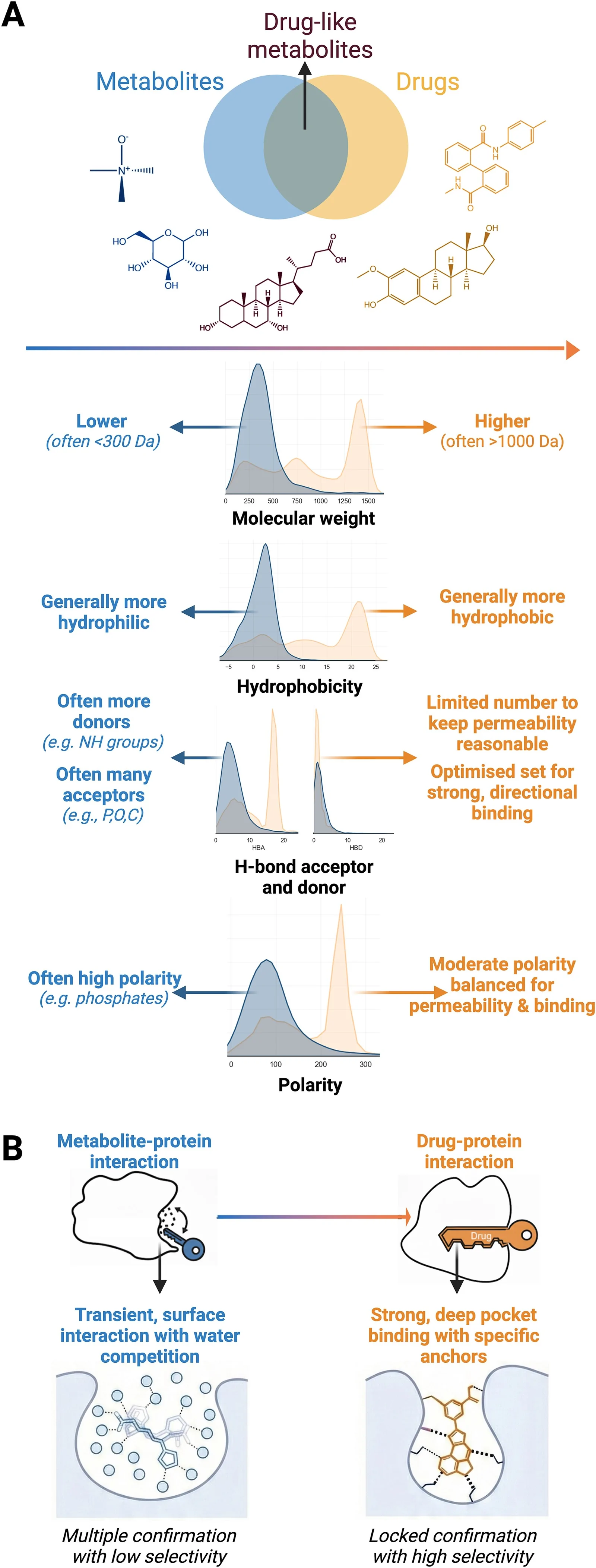
Physicochemical and binding-interface differences between metabolites and drugs. (A) Physicochemical properties of drugs and metabolites. Microbial metabolites were collected from MiMeDB (v2.0; 7 November 2025) and drugs were collected from DrugBank (v5.1.13; 8 October 2025). Both datasets were standardized, salts and duplicates were removed, and structures were adjusted to physiological pH conditions to reflect the appropriate charge. Density plots were generated from RDKit (2025.03.3) calculated properties by seaborn (0.13.2) (B) The differences between the drug–protein and the metabolite–protein interfaces.

Endogenous metabolites often occupy a different physicochemical regime from drug-oriented libraries. Many are strongly ionized at physiological pH, with high polarity and hydrogen-bonding capacity, favoring aqueous partitioning and limiting passive transmembrane diffusion. The contrast with drugs is not the mere presence of an ionizable group, but the extent and multiplicity of charge, polar surface area, and lipophilicity at physiological pH. Canonical metabolites such as carnitine, creatine, or citrate can satisfy simple count-based filters yet show poor passive permeability and rely on dedicated transport systems, while remaining unequivocally biologically active. Conversely, other metabolites (e.g. steroids, lipids) are hydrophobic and membrane-permeable, underscoring the heterogeneity of metabolite space. This mismatch in design explains why drug-trained databases and CADD methods often transfer poorly to metabolite-focused MPI discovery [126] (Fig. 2).

Physicochemical differences also shape how small molecules recognize and bind their protein targets. Drugs often occupy well-defined deep pockets with high affinity, whereas metabolites frequently bind to surface-near pockets or allosteric sites through simple hydrogen bonds or charged contacts resulting in weaker and short-lived interactions [127].

ATP binds kinases in a conserved cleft where its adenine forms hinge hydrogen bonds and hydrophobic contacts in a relatively deep pocket, while the ribose and triphosphate make polar interactions (including Mg2+-coordinated networks) that are more solvent-exposed. In contrast, many ATP-competitive inhibitors mimic the hinge interactions and, depending on class, either occupy only the adenine pocket or extend into an adjacent hydrophobic back pocket and stabilize an inactive conformation, achieving high-affinity, selective inhibition through a combination of aromatic contacts, specific hydrogen bonds, and conformational selectivity [128]. Because drug-centred tools are typically optimized to prefer rigid shape-matching and nonpolarity, they frequently fail to predict the correct pose of polar metabolites like ATP [129]. Metabolites tend to be more promiscuous because shared functional groups (e.g. phosphates, carboxylates, amines) can bind to common binding motifs across multiple proteins as cofactors, allosteric modulators, or signalling ligands, whereas drugs are optimized for selectivity [130, 131] (Fig. 2). These differences explain why successive trends of MPI methods have repeatedly needed to adapt drug-discovery tools towards metabolite-specific chemistry and context, motivating the next generation of metabolite-centred datasets and models.

### High-throughput experimental mapping of metabolite–protein interactions

Between 2010 and 2020, advances in metabolomics, proteomics, and multi-omics increased the number of robust metabolite–phenotype associations. This shifted the main bottleneck from measurement to mechanistic interpretation, identifying protein targets to regulate enzyme activity, signalling, and gene control beyond classical enzyme–substrate reactions. Earlier experimental methods mainly addressed drug-like ligands or well-studied metabolite-enzyme reactions. During this decade, new high-throughput experimental methods emerged to address this gap.

These assays fall into three categories. Metabolite-centric methods track a ‘bait’ metabolite and identify binding proteins (e.g. TPP [132], LiP-MS [133]). Protein-centric methods track a ‘bait’ protein to detect binding metabolites (e.g. DRaCALA [134]). Nontargeted methods, such as PROMIS [135], identify metabolites and proteins that co-elute after size-exclusion chromatography. These approaches have been enabled by advances in mass spectrometry, stability-based profiling methodologies, and co-fractionation [5].

Although these assays represent a significant step towards comprehensive maps of the metabolite–protein interactome, limits remain. They are most effective for stable, high-affinity interactions and struggle to detect the weak or transient interactions that constitute a substantial fraction of regulatory MPIs. Limited physiological context makes it difficult to determine whether detected interactions occur *in situ*, and several methods are prone to false positives [5]. Consequently, computational prediction and database mining remain essential for characterizing the full MPI landscape.

### From drug-centric repositories towards metabolite–protein interaction–focused resources

General CPI repositories (e.g. ChEMBL [60, 61], PubChem [36–38], BindingDB [50, 51]) continued to expand through literature curation, patents, and high-throughput screens [62]. For example, ChEMBL contained ~2 million biological activities in 2010 and >16 million in 2020 [136], reflecting a shift from small curated affinity sets to large platforms. However, these resources have remained strongly drug-focused, with endogenous metabolites representing only a minor section of the compounds. This limitation has motivated the development of metabolite-oriented resources (Table 2).

In parallel, metabolite–enzyme and transporter databases have expanded through improved curation and high-throughput methodologies, adding limited information on allosteric regulation and competitive inhibition [42, 65]. GSMMs have increased in number and coverage, with new human reconstructions such as Recon2.2, Recon3D, and Human-GEM [137–141]. Introduced in 2012, Rhea [142] shifted from a chemical-centric representation by providing expert-curated reactions whose participants are standardized to ChEBI identifiers, with explicit directionality, pH/charge states, and isomer specificity. Unlike pathway- or enzyme resources, Rhea uses balanced reactions in a standardized vocabulary, enabling comparison and linking of reactions across databases and facilitating computational integration (e.g. with UniProtKB/GO) [142]. These developments started to transform CPI and pathway resources into metabolite-centred initiatives for MPI research, contributing to the rise of dedicated MPI-aware databases in the 2020–5 era.

### Adapting CADD prediction tools to metabolite–protein interactions

CADD methodologies, initially developed for drug–protein interactions, were increasingly repurposed for MPIs. This shift reflected the growing recognition that endogenous and microbiome-derived metabolites can directly modulate host proteins, and the expansion of chemogenomic resources with large-scale compound–target annotations.

**Figure 3:**
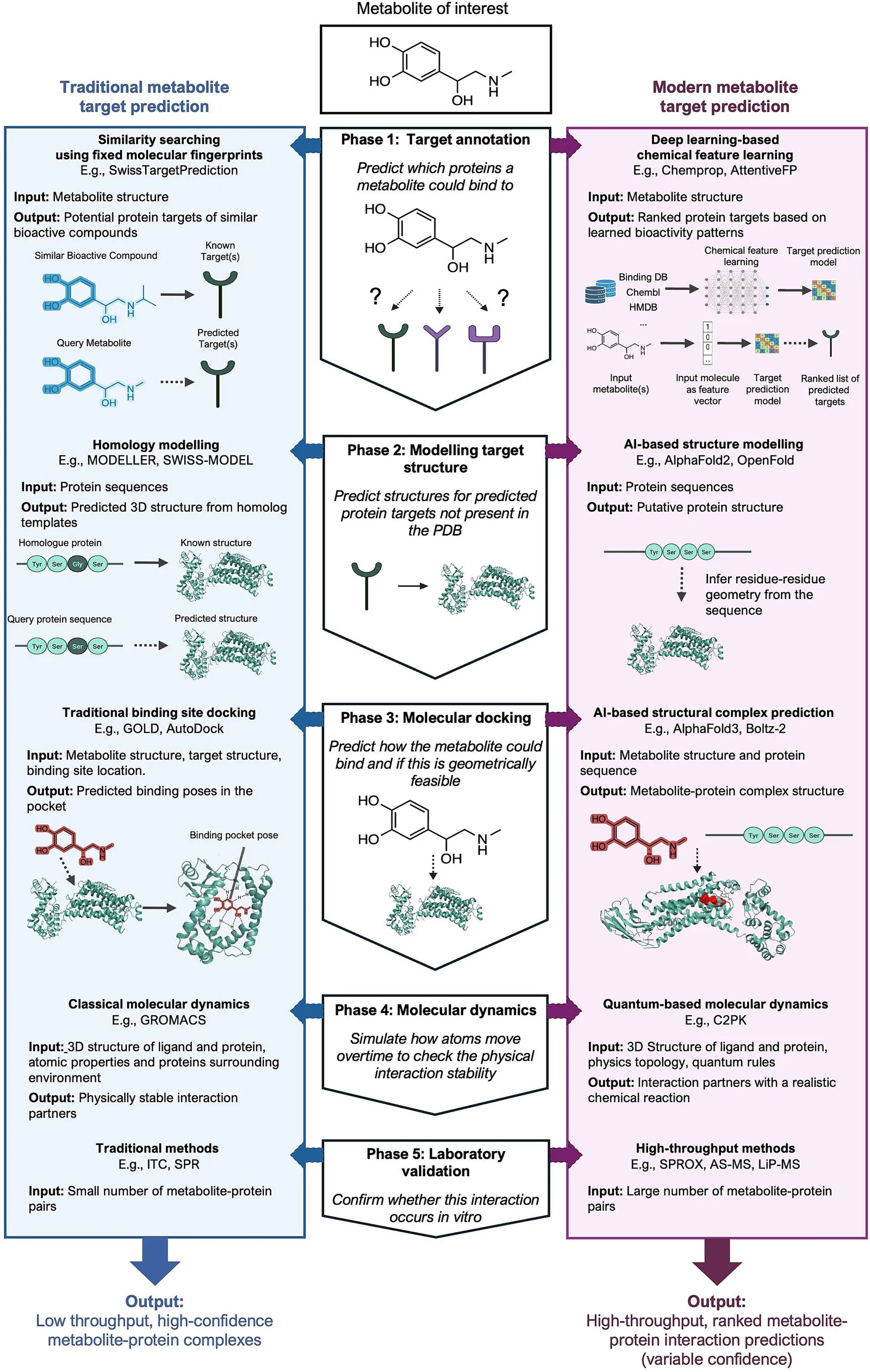
Comparative workflow for metabolite–protein target identification and validation using traditional versus modern (AI-era) approaches. A metabolite of interest is processed through five phases: (1) target annotation, (2) target structure modelling, (3) docking/complex generation, (4) molecular dynamics refinement, and (5) experimental validation. Traditional approaches (left) represent the pre-AI era and rely on similarity-, template-, and physics-based methods. Modern approaches (right) represent ~2020 onwards and use data-driven models to learn representations, rank targets, and predict structures/complexes with reduced template dependence, offering broader coverage but variable confidence that requires validation.

Traditional ligand-based target prediction represents molecules with fingerprints and infers targets by leveraging annotations from structurally similar, known ligands (Fig. 3). This similarity-driven strategy has proven effective within the well-annotated, drug-like regions of chemical space that dominate public bioactivity databases, but its performance degrades for underrepresented chemotypes and targets, reflecting dataset bias and the limits of the similarity–property principle [143]. However, many metabolites are smaller, more polar, and often exist as multiple isomers, so standard fingerprints can miss relevant variability [144]. As public resources expanded, prediction tools began mapping metabolites to targets using 2D/3D similarity (e.g. SwissTargetPrediction (2014) [145] and SuperPred (2014) [146]) while methods like OCEAN (2016) [147] combined ligand sets to infer shared targets. Alternative strategies, such as BASS [148] and BioSEA [149], compared bioactivity signatures across assays rather than relying solely on structural similarity, allowing diverse screening data to inform target prediction (Fig. 3).

By the late 2010s, the community started addressing biases derived from heterogeneous readouts and the lack of high-quality negatives. Tools such as PIDGIN [150] introduced labelled negatives and prediction confidence measures. However, these frameworks remained trained largely on drug-centric data, with limited representation of metabolite-like compounds and almost no explicit MPI datasets. As a result, 2010–20 can be viewed as a transitional phase where classical CADD target-prediction methods were adapted to metabolites but preceded the metabolite-specific resources and deep learning approaches that now enable MPI-focused modelling.

### Challenges in the 2010–20 era

Despite substantial progress, the 2010–20 period left several gaps for the next era. Available evidence was shaped by drug-centric data and by experimental constraints, leading to uneven coverage across metabolite classes, targets, and interaction types. CPI resources were dominated by drug-like ligands and enzyme targets, with relatively few records for nonenzymatic proteins like receptors and transcription factors. New high-throughput MPI experiments covered only a limited set of metabolites and proteins, constrained by the availability of tested metabolites, proteomic detectability, and bias towards stable, high-affinity interactions. Information remained scattered across multiple databases for metabolites, enzymes, transporters, pathways, and GSMMs, each with distinct identifiers and formats, and no widely adopted, central MPI repository emerged to standardize identifiers and evidence.

The 2010–20 period can therefore be viewed as a transitional phase in which scale increased faster than mechanistic certainty. High-throughput metabolomics, chemoproteomic assays, and expanded databases made it increasingly feasible to nominate candidate MPIs at system level, but interpretation remained constrained by fragmented identifiers, uneven metabolite coverage, sparse negative evidence, and weak physiological context. In practice, this era improved hypothesis generation far more than causal attribution. Its major contribution was to make MPI mapping a tractable systems problem while also revealing that increased coverage alone is insufficient without harmonization, contextual filtering, and more explicit ways to distinguish direct, indirect, and biologically plausible interactions. Altogether, MPIs remained difficult to map and interpret during this period, motivating the next phase of the field, in which harmonized databases, MPI-focused resources, and AI- or structure-driven approaches began to address these limitations.

## The era of microbiome and artificial intelligence: expanding metabolite space and metabolite–protein interaction mapping scale (2020–5)

### Microbiome-derived metabolites in metabolite–protein interaction research

Metabolomics and microbiome studies have revealed a broad landscape of small molecules arising from host biosynthesis and diet that influence health and disease. In the gut, microbes convert dietary and host compounds into metabolites that modulate host proteins and physiology. This microbially derived fraction of the metabolome includes short-chain fatty acids, bile acid derivatives, tryptophan catabolites, phenolic compounds, and other specialized microbial products [151, 152]. Early metabolomics studies have shown that microbial co-metabolites can both reflect and modulate host metabolism. In conditions such as diet-induced obesity, microbiota-dependent metabolites have been identified as predictive markers of insulin resistance and obesity [153]. Longitudinal multi-omics cohorts and consortia, including the Integrative Human Microbiome Project (iHMP) [154], MetaCardis [155], MetaHIT [156], and the Human Microbiome Standards [157] efforts and population studies such as LifeLines-DEEP [158], TwinsUK [159], and the Flemish Gut Flora Project [160], have shown coordinated shifts in microbial taxa, microbial gene content, and circulating or faecal metabolites across inflammatory, metabolic, and cardiometabolic disease states [161, 162].

The concentration and effects of microbial metabolites are highly context dependent. They vary with diet that modulates available substrates; with microbiome composition influencing metabolic pathways; with gut region, where pH differs; and with barrier integrity, which affects how much metabolites enter the tissue and blood. Many microbial metabolites are polar or ionized and thus have limited membrane permeability and complex transporter- and pH-dependent distribution; consequently, stool or plasma levels often poorly predict the concentrations at target tissues [163, 164]. They are underrepresented in drug and chemogenomic databases, often lack fully discovered structures, and only a small fraction have experimentally characterized protein targets [5]. Thus, microbiome-derived metabolites are biologically important but poorly covered by existing MPI resources [165].

With the appearance of large-scale metagenomic and metaproteomic datasets and comprehensive metabolite structure databases (e.g. HMDB, LIPID MAPs, METLIN), it has become possible to annotate metabolites by their likely source. Source-attribution databases, such as Human Microbiome-Metabolome Database (MiMeDB) [166, 167] and MetOrigin/MetOrigin 2.0 [168, 169] build on these resources by integrating structural information, enzymatic pathways, orthology, and mediation analysis to link metabolites to their microbial or dietary origins. From the genomics side, tools like PredCMB (Predicting Changes in Microbial Metabolites) [170] infer which taxa can produce specific metabolites from metagenomics data [171]. Community Spectral Libraries use untargeted MS/MS datasets to identify or classify unknown metabolites. Tools like the Global Natural Products Social Molecular Networking (GNPS) [172] and microbiome-focused tools such as the Microbe Mass Spectrometry Search Tool (microbeMASST) [173] match untargeted MS/MS spectra to reference spectra from microbial monocultures, helping to identify which strains produce a given metabolite [174] (Table 5).

**Table 5:** Resources and tools for annotating microbiome-derived metabolites and linking them to host protein targets.

**Resource (release)**	**Scope**	**Limitations**
***Annotation resources***		
METLIN [44] (2005–18)	LC-MS/MS spectral library for metabolites and small molecules with experimental and *in silico* spectra for identification	Metabolite identification without curating MPIs; limited to compounds with available spectra
GNPS [172] (2020–6)	Community MS/MS spectral libraries and molecular networking to annotate spectra and link them to known natural products	Limited to compounds with reference spectra; may miss isomers and novel chemotypes
MetOrigin [168, 169] (2021–4)	Infers metabolite origin (microbial, dietary, host, co-metabolism) using structure, pathways, and cohort mediation analysis	Dependent on database mappings; limited strain-level resolution; predictions not direct measurements
PredCMB [170] (2025)	Predicts which taxa can produce metabolites from metagenomes using pathway/orthology information	Model-based; ignores kinetics and host absorption; requires high-quality metagenomes
Microbe MASST [173] (2023)	Matches untargeted MS/MS spectra to monoculture reference spectra to propose producing taxa	Limited by available strains and growth conditions; no concentration context
***Interaction resources***		
gutMGene [176, 177] (2021–4)	Curated metabolite–host gene associations in human and mouse for gut microbe–derived metabolites	Gene-level associations; no direct protein binding or quantitative parameters
MiMeDB [166, 167] (2023–6)	Curates metabolite source attributions with linked structures, pathways, and supporting evidence	Biased to known metabolites; manual curation with limited quantitative/contextual detail
FacDB [264] (2025)	Correlates gut microbiota, gene expression, and metabolites in pigs, cattle, sheep, and chickens	Farm-animal specific; correlation-based, not validated MPIs or binding/kinetic data

Prior to 2020, the first community-scale analyses of how microbial production and consumption of metabolites shape host cell environments were published (e.g. the NJS16 network [175]), although host nodes were defined at the cell-type rather than protein or gene level. From 2020 onwards, databases such as gutMGene [176, 177] and MiMeDB [166] have extended this paradigm by adding host protein targets and richer contextual annotations. gutMGene was the first curated database to provide systematic maps of microbial metabolite–host gene interactions, while MiMeDB further annotated many microbial metabolites with human protein targets and bioactivities. In parallel, metabolite-focused interaction and communication resources (e.g. ligand–receptor and MPI databases and knowledge graphs) can be intersected with these microbial metabolite catalogues to identify candidate microbial metabolite–host protein interaction networks and metabolite-driven cell–cell communication maps (Table 5).

For most microbiome-derived molecules, we still lack quantitative exposure data at the relevant host tissues, knowledge of the transporters that move them across barriers and into cells, and, most importantly, systematic maps of their host targets https://sciwheel.com/work/citation?ids=16579519&pre=&suf=&sa=0 [178, 179]. Addressing these gaps requires metabolite-focused ligand- and structure-based models, integrated with source attribution, transport, and host expression data, to prioritize microbial MPIs that are not only chemically but also physiologically feasible, as testable hypotheses rather than final solutions.

### Rise of dedicated metabolite–protein interaction–focused resources

In parallel with the microbial source-attribution and metabolite-annotation tools, MPI-focused resources have increasingly been published to discover nonenzymatic targets. This growth reflects two converging pressures, the expanding interest in metabolites as regulators and signalling molecules, and the need for searchable, harmonized evidence to support systematic inference and benchmarking.

A first group includes ligand–receptor databases, developed for analysing cell–cell communication. These are manually curated, relatively small resources (~100–1000 interactions) between small molecules such as metabolites or protein ligands and receptors (e.g. MRCLinkDB, MEBOCOST, CellChatDB [180–182]). Their developments have grown with the increasing use and development of single-cell transcriptomics [183, 184]. However, their narrow scope overlooks the hundreds of metabolites not studied as extracellular signalling molecules [180, 185] (Table 2). Whilst cell–cell communication databases focused on curated metabolite–receptor interactions; a second group includes integrative MPI hubs that not only include cell–cell communication knowledge but also integrate broader MPI evidence. MetaLinksDB [186] was the first attempt, integrating data from STITCH [57–59], Rhea [142], GSMMs, and cell–cell communication databases. Because most associations derive from STITCH, which has not been updated since 2016, MetaLinksDB inherits STITCH’s dated and incomplete MPI coverage (Table 2). A third group directly captures outputs from high-throughput interaction screens. PMI-DB [187] is the first resource to systematically collect MPIs from LiP-MS, TPP, and related assays, assembling ~50k curated MPIs for 23 small molecules across four species, and, unusually, including negative interactions (Table 2).

These resources mark an important shift from generic, drug-centred CPI repositories to MPI-aware databases, but at present, they function more as complementary evidence layers than as comprehensive stand-alone resources [5, 188]. Their current importance lies less in completeness than in making MPI evidence more searchable, harmonizable, and reusable for downstream prediction and benchmarking. Further development should increase their impact on MPI prediction and integration methods.

### Modern metabolite–protein interaction prediction: towards metabolite-aware artificial intelligence models

While earlier computational work largely adapted classical drug–target discovery pipelines to metabolites, the 2020–5 era has seen the appearance of metabolite-focused AI-driven models for MPI prediction. This shift was enabled by increased availability of MPI-centric resources (e.g. PMI-DB [187]) alongside modern molecular representations and deep learning architectures (e.g. attention- and graph-based models [189, 190]). However, more advanced models do not automatically remove the underlying biological uncertainty. Based on the current literature [191, 192], AI-based approaches are most useful for prioritizing a wider set of candidate MPIs, while distinguishing interactions that are structurally plausible from those that are biologically relevant remains a major challenge.

Within this period, progress followed several distinct, partly overlapping trends. Firstly, ligand-based prediction tools have shifted from fixed descriptors towards deep learning approaches such as Chemprop [193] and AttentiveFP [189] (Table 3). Instead of relying only on pre-computed fingerprints, these graph-based models learn molecular features directly from structure. In parallel, fingerprints themselves also evolved. New designs aimed to represent the small, polar, and structurally diverse metabolites improving similarity search and classical ML performance [194, 195], including MAP4 [196] and MHFP [197]. Thirdly, pipelines more often adapted similarity and graph-neural network (GNN) models on MPI-relevant datasets with greater emphasis on label harmonization [195] and recorded negative cases while continuing to use classical similarity tools as baselines or in consensus [150]. Finally, complementary research began to address the shift in drug-to-metabolite chemical space. For example, Wu *et al.* proposed a deep-learning framework that transfers general interaction patterns from well-studied chemical space (e.g. drug–protein interactions) to less studied areas such as MPIs. Whilst this leads to a large improvement in the ability to predict human MPIs, there will still be false positives requiring further validation [198].

Structure-based approaches remain essential for MPI prediction but have been transformed by proteome-scale deep learning models. AlphaFold (AF) [191, 199–201] RoseTTAFold [202], ESMFold [203], and OmegaFold [204] provide near proteome-wide coverage of single-chain protein structures. The AlphaFold Protein Structure Database offers curated, searchable access to these modelled structures at scale [200, 201], whilst OpenFold [205] and ColabFold [206] improve accessibility. These models learn protein folding from evolutionary sequence variation in multiple sequence alignments and structural rules from training structures in the PDB. This allows the generation of highly accurate 3D structures for many targets that previously lacked experimental models. More recently, AlphaFold-Multimer predicts multi-chain complexes, contextualizing active and allosteric sites in oligomeric proteins [207]. This removes a major bottleneck for docking and pocket analysis, which previously relied on classical homology modelling [103, 105] (Table 4, Fig. 3). However, these advances have not eliminated classical homology modelling, which remains useful when the model confidence is low (e.g. flexible/disordered regions) and when a modeller needs to enforce a specific experimentally observed conformation by choosing a particular template [208]. Overall, deep learning has shifted homology modelling from a primary route to a complementary/backup strategy, rather than fully replacing it.

More recent deep-learning models directly predict metabolite–protein complexes. AlphaFold3 (AF3) [191] and RoseTTAFold All-Atom [209] predict assembly of proteins with small molecules, ions, and nucleic acids, thereby offering candidate MPIs. Although AF3 is the most prominent tool, achieving 76% accuracy on the PoseBusters benchmark [191], assessing its utility for MPIs is challenging. PoseBusters is a widely used benchmark for drug-like interactions by using high-resolution (<2.5 Å) post-2021 PDB structures and strict physical validation checks [192]. However, this dataset is biased towards rigid, high-affinity drug inhibitors. Consequently, the benchmark is less applicable to MPIs, as metabolites often bind flexibly and transiently, forming dynamic transition states that are poorly represented by static crystal structures (Fig. 3).

There are a few studies already demonstrating limitations in AF3’s MPI predictions. Performance is accurate for rigid saturated fatty acids but notably degrades for unsaturated and branched-chain fatty acids [210]. Although AF3 predicted structurally feasible glycan–protein complexes, it did not represent the conformational dynamics that often drive glycan recognition and mechanism [211]. Furthermore, accuracy declines significantly when backbone displacement exceeds 5 Å, a common occurrence in enzymatic catalysis [212]. This demonstrates that AF3 can model certain MPIs successfully but struggles with interactions that are not covered by its training data.

There are several deep-learning structural prediction models that have attempted to address the remaining gaps, particularly around more realistic binding, conformational change, and small-molecule interactions. Chai-1 builds on a protein-language model trained on the PDB dataset but allows the user to add prior knowledge, such as expected residue–ligand contacts or interaction geometry, to guide the prediction process [213]. One year later, Boltz-2 was published to address previous limitations in knowledge of rigid structures and binding strength. The tool takes a different approach by incorporating binding affinity and short MD trajectories alongside PDB structural data. MD improves the prediction of MPIs, as PDB entries are often biased towards apo (open) or inhibitor-bound states and lack the transient conformations required for metabolite binding [214]. In parallel, efforts such as NeuralPLexer (NP3) retained PDB-scale training but shifted the strategy towards physical validity and conformational diversity, prioritizing binding pockets, interfaces, and distributions over single static models. By generating structural ensembles, NP3 can capture distinct bound and unbound states, making it more accurate for modelling motions >5 Å, frequently encountered in enzymes and transporters during metabolite binding. This is illustrated by predictions in which tryptophan binding induces conformational changes shifting the protein between distinct open/closed states [215]. These models all have similar accuracy to AF3 on the PoseBusters datasets, but as previously mentioned, this benchmark is less useful for metabolites as the dataset reflects the drug-like molecule bias of the PDB [192].

In summary, the 2020–5 era moved MPI prediction from adapted drug-discovery workflows towards metabolite-aware, structure-guided AI, enabling broader *in silico* screening while shifting the remaining bottleneck to context, benchmarking, and validation.

### Convergence of omics in metabolite–protein interaction research

A major driver for mapping MPIs is the need to integrate metabolomics with other omics layers in order to link metabolic changes to downstream molecular mechanisms. Multi-omics studies aim to explain disease pathogenesis and cellular states by combining metabolomics with transcriptomics, proteomics, or epigenomics. Broadly, integration strategies fall into two categories, data-driven and knowledge-based approaches [216].

Data-driven methods, such as Multi-Omics Factor Analysis (MOFA and MOFA+) [217, 218] use statistical modelling and dimensionality reduction to identify correlations or latent factors across omics layers. These approaches can reveal patterns without prior assumptions but are agnostic to mechanism and typically require pathway enrichment or network analysis for interpretation [219].

Knowledge-based approaches integrate omics readouts with prior networks of known interactions, including metabolic reactions, protein–protein interactions, and gene regulatory interactions. Tools like COSMOS [138] use constraint-based network propagation to combine prior knowledge with multi-omics. For MPIs, these networks provide the missing edges that connect metabolites directly to proteins, linking metabolite changes to signalling and gene regulation [138]. Complementary approaches such as MetaboSignal construct integrated metabolite–gene networks by merging KEGG metabolic and signalling pathways, enabling topological analysis of how changes in metabolite levels are linked to regulatory genes and pathways defining metabolic signatures [220]. However, prior-knowledge networks are incomplete, biased towards well-studied pathways and drug targets, and often lack cell type or condition specificity [221]. This dependence shows the need for MPI resources that systematically curate metabolites, including diet- and microbiome-derived compounds, and provide context-specific modelling.

### Remaining challenges in the artificial intelligence era

By 2025, MPI research had made progress in reducing data fragmentation and protein structure availability, but challenges about reliability and bias became increasingly apparent. Experimental evidence remains dominated by drug-like ligands and well-studied protein families while high-throughput assays (e.g. TPP [132], LiP-MS [222], PROMIS [135]) cover only selected metabolites and conditions and rarely report affinities or *in vivo* context.

On the modelling side, deep learning models enabled structure and complex prediction at a scale that would have been unrealistic a decade ago [191, 209]. Yet benchmarks suggest that improved pose generation does not consistently translate into better virtual screening. In many settings these models still struggle to distinguish binders from nonbinders because binding affinity is poorly captured [192, 223]. Critical evaluations further show that co-folding models can produce physically unrealistic geometries, reflecting training data bias and incomplete learning of the underlying physical constraints on molecular interactions [224]. Thus, current models often demonstrate structural feasibility without establishing thermodynamic or physiological context. This distinction is critical in practice; just because an interaction appears structurally plausible does not mean it is mechanistically relevant in a biological context. A useful MPI workflow must therefore treat AI predictions as one evidential layer among several, to be interpreted alongside localization, abundance, transport, and experimental target engagement data.

Data bias remains central, most predictors are trained mainly on drug–target interactions, and can underperform on smaller, more polar, and highly charged metabolites [122]. Co-folding models are primarily trained on PDB data, which are biased towards well-studied, high-affinity interactions and may not generalize to weaker, transient MPIs. This also complicates model evaluation. Posebusters is a strong standard for drug-like molecules but is less informative for metabolites because it reflects the same structural and chemical biases [192]. Progress will require metabolite-focused benchmarks including chemical heterogeneity and multiple conformational states.

Robust training is further limited by scarce, well-defined negative data, increasing the risk of geometrically plausible but biologically irrelevant predictions [225]. Methods that model physical constraints, such as NP3, can mitigate failures, but negative matches would allow models to learn to penalize false-positive interactions more effectively [215].

Finally, most of the models do not capture the biological context. Metabolites and proteins must co-localize in space and time, be present at sufficient concentrations, and remain accessible given transport and localization barriers. Native protein state, including complexes, cofactors, and post-translational modifications, can further allow or prevent binding. Without integrating this information, structurally plausible predictions may not be relevant biologically.

The 2020–5 era did not remove the core uncertainty in MPI research but rather relocated it. Earlier periods were limited mainly by sparse data and incomplete structural coverage; the AI era greatly expands candidate generation and structural hypothesis space yet shifts the main bottleneck towards calibration, benchmarking, and biological interpretation. Modern models are increasingly effective for prioritizing candidate MPIs, but they are not yet equivalent to mechanistic evidence. The practical consequence is that MPI workflows must now be designed around evidence integration: combining prediction, context filtering, and targeted validation, rather than treating any single model or assay as sufficient on its own.

Across these historical periods, MPI methods improved along three partially independent axes: scale, structural resolution, and integrative capacity. Single-target biochemistry and structural biology provided the strongest mechanistic confidence but limited coverage; high-throughput metabolomics and chemoproteomics expanded discovery but often with weaker causal resolution; and recent AI- and structure-driven methods now allow many more candidate MPIs to be prioritized, while increasing the need for careful benchmarking and context-aware filtering. A useful way to interpret this progression is that different approaches contribute distinct evidence: some generate hypotheses, some provide direct interaction evidence, some establish physiological plausibility, and only a subset supports causal inference. This distinction is important in practice because robust MPI interpretation depends less on any one tool than on how these layers are combined.

## Future outlook: from harmonized data to contextualized deep-learning and systems-level networks

The historical trajectory of MPI research shows a recurring trend: our ability to detect metabolite associations has grown rapidly, yet mechanistic interpretation remains limited. The next phase should therefore not simply expand interaction lists, but build on causal, context-aware MPI maps, supported by standardized evidence, improved measurements, and modern inference frameworks that consider multiple parameters rather than affinity alone [226, 227]. In practice, this requires a shift from asking whether a metabolite can bind a protein under any condition to asking when that interaction is sufficiently supported, contextually feasible, and functionally relevant to inform biological interpretation.

A useful way to guide future MPI interpretation is to distinguish between three linked evidence layers: binding plausibility, contextual feasibility, and functional consequence, while considering uncertainty at each step. Structural or ligand-based predictions can support binding plausibility, but they do not establish that a metabolite reaches the target in the relevant compartment or concentration range. Proteome-wide target engagement assays can support physical interactions in a given condition, but they do not by themselves demonstrate downstream functional impact. Similarly, functional readouts alone may show that a metabolite affects a pathway or phenotype, but without direct binding evidence, they may not identify the specific protein target responsible. Future progress will therefore depend less on any single technology than on frameworks that integrate this evidence explicitly while reporting the confidence and limitations associated with each one.

From this perspective, MPI methods are best viewed not as competing alternatives but as tools suited to different types of interpretation. Curated databases and ligand-similarity approaches are most useful for hypothesis generation, especially when a metabolite lies near previously annotated chemical space. Structure-based modelling is most useful for assessing binding plausibility once candidates have been prioritized. High-throughput experimental methods are most useful for discovering condition-specific target engagement, but their outputs require careful interpretation because detected signals may derive from direct binding, indirect pathway effects, altered protein complexes, or changes in protein stability rather than a single direct MPI. Context-resolved readouts, including transporter information, subcellular localization, abundance, and spatial or single-cell profiling, are essential for evaluating biological feasibility. Finally, perturbation-based experiments are required to establish functional and causal relevance. A practical future direction for the field is therefore not a one-step prediction framework but a staged evidence-integration strategy in which different methods contribute different kinds of support.

### Short-term goals: turning interaction lists into systems-level mechanistic maps

Over the next 1–3 years, the field’s most realistic priority is to make existing and emerging evidence sufficiently standardized, comparable, and context-resolved for mechanistic interpretation. Historically, progress has been limited less by the lack of discoveries than by fragmented evidence and inconsistent metadata, which directly hinders reproducibility and weakens *in silico* model training and benchmarking. MPI resources should therefore clearly distinguish predicted interactions, experimentally detected interactions, and experimentally supported interactions with known biological context, such as tissue, cell type, compartment, concentration, or target expression. This is essential for understanding how metabolites affect cellular behaviour at the systems level, rather than continuing to accumulate poorly comparable interaction lists.

#### Harmonize identifiers, metadata, and evidence

A key opportunity is to build central, standards-driven MPI databases that precisely define metabolites, collect high-quality interactions, and integrate them with existing metabolite, chemical, and structural resources (e.g. ChEBI, HMDB, and the PDB) and biological context. MPI evidence accumulated slowly through targeted biophysical and enzymology studies (e.g. SPR, ITC) and therefore remains widely fragmented [5]. More recently, proteome-wide assays such as TPP or LiP-MS have introduced a new trend by generating large, quantitative MPI evidence together with experimental context (e.g. concentrations, sample type, replicates) that can be captured as assay metadata, including matched negatives. However, results often remain scattered across publications and local repositories. Establishing minimal reporting standards for MPIs (metabolite origin, target protein isoforms, and specific biochemical mechanisms) would allow coherent data aggregation across studies and improve the interpretability of downstream analyses [63].

To support reliable AI models, repositories should also curate matched negatives (e.g. analysing through the same assay type and conditions). These negatives are essential as training sets for AI models, enabling calibrated confidence scores and reducing false positives [150, 187]. Curated negatives should be annotated with the assay’s sensitivity and range (e.g. concentration window, temperature, pH, compartment), so models learn context-specific noninteractions rather than defining the universal absence of binding [63, 150, 187]. Because computational predictions will continue to fill experimental gaps, databases should include *in silico* entries with clear labelling (algorithm and version, and any physiological filter (e.g. location or concentration) applied). Experimental and predicted evidence should remain distinct but interlinked, so users can filter by evidence type [186, 187].

#### Increasing physiological context through spatial and *in situ* measurements

The value of a comprehensive MPI database depends on experimental coverage and physiological relevance. Therefore, such a database should detail the tissue and cellular localization of metabolites and their protein targets, providing the biological context necessary to evaluate whether interactions will occur *in vivo*. An important shift that has already begun is moving metabolomics from bulk biofluids (blood, faeces, urine) towards tissue-derived measurements. While traditional metabolomics provide limited information about where metabolites act in tissues, tissue metabolomics detect metabolites within the relevant anatomical site, improving inference about whether metabolites are likely to be the affected cells [228].

Spatial metabolomics approaches, such as MALDI-MSI and DESI-MSI [229, 230], provide a map of metabolite distributions and are most informative when integrated with spatial or single-cell transcriptomic/proteomic readouts that identify the cell types and target proteins present in the same regions. By combining metabolite hotspots with local target expression, co-localization can be assessed at the microenvironment level. When a metabolite signal and its putative target are supported to co-occur in the same niche, database- or model-derived MPIs become more feasible *in situ* and can be prioritized for targeted validation and downstream network modelling [231, 232].

#### Move towards metabolite-centred machine-learning training and evaluation

A major short-term modelling priority is to move further away from models primarily trained on drug-discovery datasets. Over the next few years, better-labelled MPIs from proteome-wide chemoproteomic and structural proteomic assays should support predictors that learn metabolite-like binding behaviour and pocket geometry, rather than inherited biases from drug discovery [191]. Progress will also depend on a metabolite-focused benchmark. Without evaluation sets that reflect the diversity of the metabolome, results will remain difficult to interpret [233]. Given the heterogeneity of the metabolome, a practical strategy is to develop benchmark subsets by metabolite class (e.g. lipids, carbohydrates, co-factors) clarifying which interaction types are currently predictable and where the performance is low. Reducing drug bias in training data will require metabolite-specific representations and attention to methods that allow pocket flexibility rather than a single rigid site [234].

At the same time, the field is increasingly influenced by multi-modal and multi-view learning, in which models combine multiple input types such as protein sequence/structure features and molecular representations. For metabolites, integrating complementary encodings (e.g. fingerprints, graph features, and SMILES-derived embeddings) can be more robust than relying on a single description, particularly when curated MPI labels remain sparse. Recent work that combines classical chemical features with transformer-derived molecular embeddings illustrates how combining chemical knowledge with learned representations can improve performance [235]. For MPI prediction, these designs should be tested on metabolite-relevant benchmarks and paired with clear applicability domains and calibrated confidence, hence increasing scale and improving coverage without introducing more false positives.

#### Embedding metabolite–protein interactions into network and constraint-based models for mechanistic modelling

MPIs can provide mechanistic edges connecting metabolite state to enzyme activity, signalling, and gene regulation, helping convert metabolomics associations into causal hypotheses [220]. The short-term opportunity is not only to predict more MPIs but also to interpret them through models that explicitly encode biological feasibility, such as whether a metabolite can reach a target at a relevant concentration, in the correct compartment, and in a condition where the target is present and functionally accessible [236, 237].

For constraint-based and network models, this motivates adding context constraints, not only stoichiometry or topology. In GSMMs, MPIs can be represented as regulatory constraints on reaction capacity, combined with transporter- and compartment-aware accessibility, and condition-specific expression, so that predicted flux states respond to metabolite perturbations in a biologically realistic way. More generally, the same context layers can improve any MPI-informed modelling framework, including signalling networks, causal graphs, or hybrid ML-based pipelines, by filtering interactions and weighting edges according to plausibility [238, 239].

Crucially, many of the required constraints are already measurable or inferable from existing data types, such as metabolite availability from (tissue/spatial) metabolomics [232]; target presence and cell-type specificity from bulk or single-cell transcriptomics and proteomics [240]; compartment localization and transport capacity from transporter annotations and localization resources [75]; and protein state from phosphoproteomics/PTM profiling [241] and target engagement assays such as TPP or LiP-MS [132, 242].

Achieving this will require benchmarks that link metabolite measurements, context variables, protein state readouts, and phenotypic outcomes, alongside iterative feedback where validated MPIs update shared resources and refine system-level models over time.

#### Systems-level integration: the host–microbe paradigm

**Figure 4:**
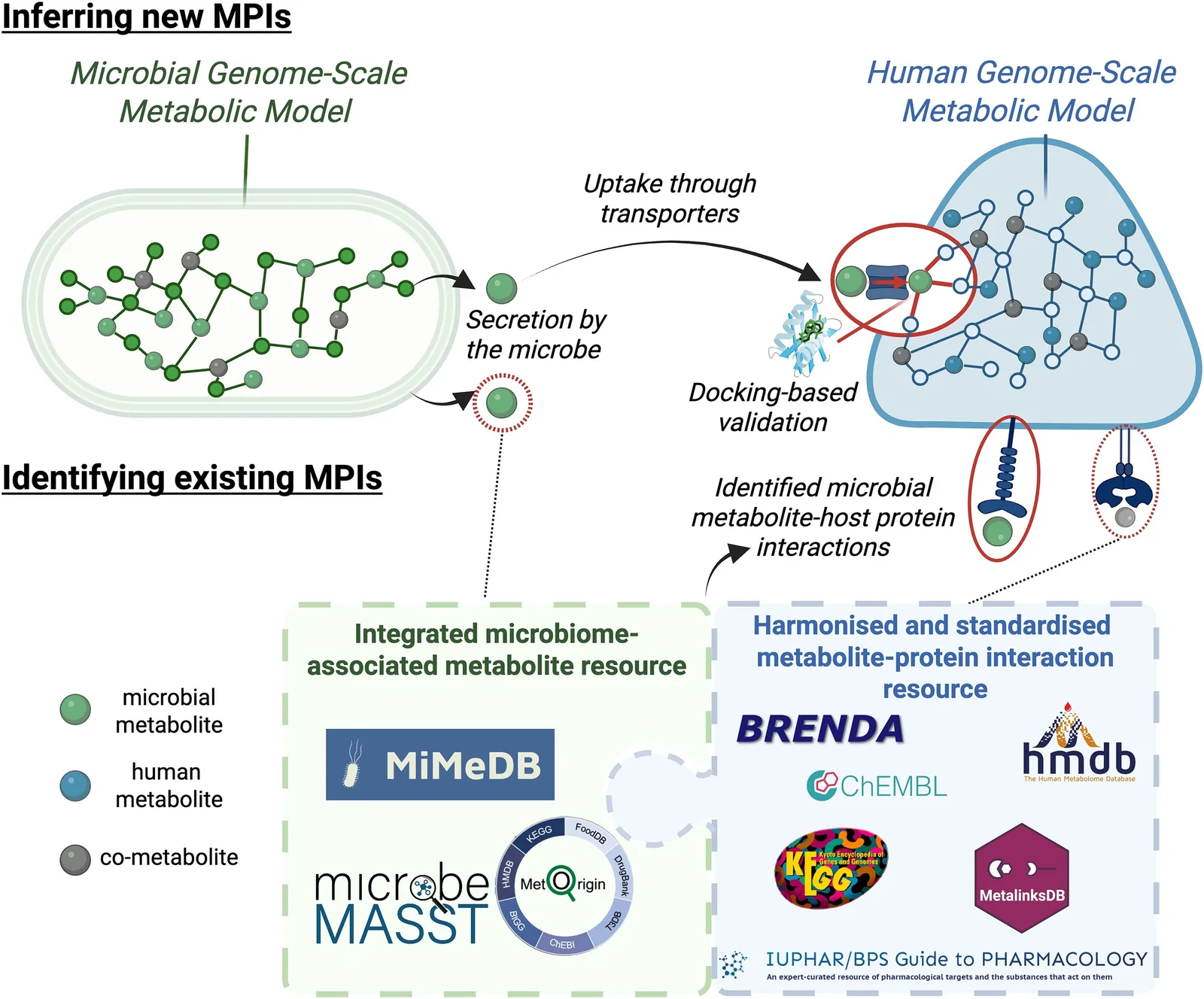
Integrating (spatial) metabolomics with (single-cell/spatial) transcriptomics to map microbial metabolite–host protein interactions. The upper section illustrates inferred MPIs generated by combining microbial and host genome-scale metabolic models (GSMMs) with *in silico* docking to prioritize candidate host targets. The lower section summarizes designated microbiome-associated metabolite resources and MPI databases used to identify existing microbial metabolite–host protein interactions.

The greatest advantage of these historical advances in MPI research is realized in complex systems biology, with host–microbiome research providing a great application area. A future interspecies MPI workflow can build an interpretable causal chain by identifying microbiome-derived metabolites using source-attribution databases, modelling their production and exchange via GSMMs (e.g. AGORA2 [243], Recon3D [141]), and applying transporter maps to refine tissue and compartment accessibility [244]. The resulting metabolite set can then be intersected with centralized MPI resources to generate a metabolite-mediated host–microbe interaction layer. In parallel, protein-mediated interkingdom communication can be inferred from microbial proteins using prediction tools such as MicrobioLink [245], which predicts host–microbe protein–protein interactions and explores their potential downstream effects on host signalling. Together, these complementary layers support a more complete and testable model of how microbial variation perturbs host pathways through both metabolites and proteins. Importantly, this does not identify targets by itself, but it narrows the hypothesis space to metabolites that are both producible and biologically accessible in the relevant context (Fig. 4).

Within this constrained set, candidate host targets of these metabolites can then be prioritized *in silico* through structure-aware predictions to suggest binding modes and rank proteins by interaction likelihood [225]. Finally, targeted assays can test these hypotheses, providing an experimental path from metabolite exposure to target engagement and downstream phenotypic effects [132, 133] (Fig. 4). Combining these steps has the potential to move the field from interaction lists to context-filtered, testable MPI networks, enabling more precise target identification and supporting metabolite-based intervention and repurposing strategies [246].

### Long-term challenges: from mechanistic metabolite–protein interactions to complete cellular modelling/predictive systems biology

In the longer term, progress will depend on moving beyond interaction-centric descriptions towards predictive frameworks that distinguish structural possibility, biological feasibility, and functional consequence. A metabolite may be able to bind a protein *in silico* or *in vitro* without exerting a measurable effect in the relevant tissue, time window, or cellular state. For this reason, future systems-level MPI models will need to incorporate not only binding hypotheses but also competition, compartmentalization, protein state, transport constraints, and downstream functional readouts. At present, however, most of this information is unavailable for the vast majority of MPIs. Metabolite concentrations are often measured in bulk rather than in the relevant compartment, transporter dependencies are incompletely annotated, protein state is highly condition-specific, and downstream functional effects are difficult to assign to a single MPI. The major challenge is therefore not simply to predict more interactions, but to learn when a predicted interaction becomes mechanistically relevant. Once MPI evidence becomes broader and better benchmarked, the long-term challenge shifts from target identification to predicting functional outcomes in complex biological systems. The likely next trend is a transition from interaction-centric views to modelling how MPIs reshape metabolism, signalling, and disease mechanisms in dynamic biological systems.

#### Completing the metabolite space: the ‘dark metabolome’ as a ceiling on systems models

Systems-level MPI modelling is limited by incomplete metabolite identification. A major fraction of LC-MS features remain unidentified, described as the chemical ‘dark matter’ of the metabolome [247]. This is particularly limiting the understanding of host–microbe interactions, where metabolite diversity is high. Long-term progress therefore depends on improved identification pipelines (better annotation, spectral/retention-time prediction, and sensitivity for low-abundance features), so models are built on a more complete and interpretable set of metabolites [248].

#### From static binding to functional outcomes in dynamic systems

Long-term progress will require models trained and evaluated on metabolite-like binding behaviour, including weak, transient, and allosteric interactions [249]. Recent structure-aware foundation models (e.g. Boltz-2 [214]) represent early steps towards incorporating affinity-related objectives and information relevant to conformational dynamics.

However, dynamics and atomistic accuracy do not resolve the central MPI challenge of context-dependent binding. Whether an interaction is biologically relevant depends on exposure and access, such as metabolite concentrations [250], compartmentalization [251], transporter-mediated accessibility [252], competition with endogenous ligands [249], and whether metabolic flux permits meaningful target engagement. Predictive frameworks should therefore combine binding hypotheses with biological context filters rather than treating binding as a context-independent property of a protein–ligand pair.

The current interaction evidence is biased towards stable, assay-based interactions, which can underrepresent short-lived, state-dependent binding [253]. Addressing this mechanistic gap will likely require combining conformational ensembles and transient contacts via MD simulations [254], complemented by energetics-based refinement hybrid quantum mechanics/molecular mechanics (QM/MM) such as CP2K [255–257], and ML-based quantum models such as OrbNet Denali [258], to connect binding to mechanism, particularly where pocket opening or allosteric regulation is central. In the short term, these tools are most realistic as refinement for high-priority candidates, but over time, improved efficiency and better integration into large scale pipelines may allow broader use.

#### Training-data limitations: how to teach models about dynamics and weak binding

Most labelled MPI datasets overrepresent static, stable interactions, so models trained on them tend to underperform on transient, context-dependent binding. Simulation-derived labels are uncertain and must be treated as hypotheses. The long-term progress will require community benchmarks that explicitly score models on weak/transient interactions, competition/allostery, and transfer across cellular states, alongside calibrated uncertainty so predictions remain interpretable rather than overconfident [192]. A potential complementary approach is chemical trapping strategies such as photoaffinity labelling, which can freeze short-lived interactions in cells and thereby expand the observable interaction space. The limitation of this is that probe design can perturb native binding [259].

## Conclusion

Over the past decades, MPI research has shifted from fragmented, drug-centred datasets to systematically curated resources and increasingly integrative, model-driven interaction maps. Yet, without mechanistic links to the cellular functions that sense and execute metabolite signals, these associations often remain descriptive. Closing this gap is essential for converting metabolomics from profiling into a causal framework that can explain phenotypes, prioritize interventions, and support translation.

The expansion of high-throughput metabolomics, chemoproteomic assays, curated MPI resources, and AI-based prediction has transformed what can be studied at the systems level. However, it has also made interpretation more difficult: coverage has increased faster than our ability to distinguish direct from indirect, plausible from implausible, and structurally possible from biologically meaningful MPIs. The central task of the next decade is therefore not only to accumulate more interactions but also to organize them into a mechanistically interpretable evidence framework.

Looking ahead, three broad priorities and a set of key application areas are likely to shape how the field develops over the next decade. Firstly, MPI data need to become more complete and more comparable across systems. Systematic curation of metabolites from host, diet, and microbiome, recording assay type, directionality and mechanism, and harmonized structural identifiers will determine the reliability of downstream models. Second, predictive models should become explicitly metabolite-focused. As long as models rely primarily on drug–target datasets, they will underperform on small, polar, charged metabolites and on weak or transient interactions. Progress will therefore require training and benchmarking on curated MPI datasets, supported by proteome-scale structures and integrated use of docking and simulations, to move from ‘repurposed drug tools’ to MPI native prediction tools. Third, predictions must be interpreted through physiological context. Spatial and single-cell omics, transporter maps, and GSMMs provide complementary constraints for deciding which predicted MPIs are likely to occur *in vivo*.

Several application domains can benefit directly from these developments. In network biology, integrating MPIs into GSMMs and constraint-based flux analyses will allow metabolite changes to be connected to pathway activity and cross-talk between metabolism and signalling. In nutrient sensing and metabolic signalling, MPI maps can clarify how small molecules regulate kinases, GPCRs, nuclear receptors, and other sensors that link metabolic state to transcription programmes, cell growth, and inflammation, directly informing drug discovery on these target classes. Finally, many post-translational modifications are directly limited by MPIs, because their donor groups are metabolites or metabolite-derived cofactors. Systematic MPI resources can help to link metabolite availability to the regulation of chromatin-associated proteins, signalling molecules, and structural proteins, offering a mechanistic route to understand how metabolic state defines cell identity, signal integration, and the robustness of cellular responses.

Despite the rapid progress in databases, technologies, and AI models, a large part of the metabolome remains uncharacterized, known MPIs are sparse, and current models are not sufficiently accurate to act as ground truth. Experimental approaches that directly measure interactions, conformational changes, and functional outcomes will therefore remain essential. A realistic next step is to adopt an iterative framework in which harmonized MPI resources support better models; models generate context-aware hypotheses; and targeted experiments feed refined evidence back into shared databases. If the field can combine high-quality, standardized data with metabolite-focused models and physiological context, MPIs should over the next 10 years evolve from scattered interaction lists into robust, causal frameworks that support network modelling, clarify metabolite-driven signalling, and enable metabolite-centred therapeutic strategies.

**Key Points**

- Metabolite–protein interaction (MPI) research has progressed from low-throughput, high-confidence studies of selected targets towards increasingly scalable but methodologically heterogeneous approaches that require careful interpretation.
- Early drug-centric resources and computational models historically limited MPI discovery because endogenous metabolites differ fundamentally from drugs in chemistry, binding behaviour, and physiological context.
- The 2020–5 era expands MPI mapping through microbiome-derived metabolite research, improved metabolite annotation, and new MPI-specific resources that integrate experiments with computational tools.
- Modern deep-learning models and structural predictors (e.g. AlphaFold3, RoseTTAFold All-Atom) enable metabolite-aware target prediction but remain challenged by data bias, lack of negative interactions, and metabolite-specific complexities.
- Future progress requires centralized MPI databases, standardized data, and context-aware prediction frameworks that integrate multi-omics, spatial information, and physiological constraints to generate mechanistic, testable hypotheses.

## Abbreviations

ADME, Absorption, Distribution, Metabolism, and Excretion; AF, AlphaFold; AGORA2, Assembly of Gut Organisms through Reconstruction and Analysis 2; AI, artificial intelligence; Å, Ångström; ATP, adenosine triphosphate; BLAST, Basic Local Alignment Search Tool; Boltz-2, not expanded in text (model/tool name); CADD, Computer-Aided Drug Design; CAS, Chemical Abstract Service; ChEBI, Chemical Entities of Biological Interest; COSMOS, Causal Oriented Search of Multi-Omics Space; CPI, chemical–protein interaction(s); Da, dalton; DESI-MSI, Desorption Electrospray Ionization Mass Spectrometry Imaging; DNA, deoxyribonucleic acid; DRaCALA, Differential Radial Capillary Action of Ligand Assay; EMBL-EBI, European Molecular Biology Laboratory—European Bioinformatics Institute; FFAR2, Free Fatty Acid Receptor 2; FFAR3, Free Fatty Acid Receptor 3; FXR, Farnesoid X Receptor; GC-MS, Gas Chromatography–Mass Spectrometry; GNN, graph neural network; GNPS, Global Natural Products Social Molecular Networking; GO, Gene Ontology; GPCR(s), G-Protein Coupled Receptor(s); GPR41, G-Protein Coupled Receptor 41; GPR43, G-Protein Coupled Receptor 43; GSMM, Genome-Scale Metabolic Model; HMDB, Human Metabolome Database; IBD, Inflammatory Bowel Disease; iHMP, Integrative Human Microbiome Project; InChI, International Chemical Identifier; ITC, Isothermal Titration Calorimetry; KEGG, Kyoto Encyclopedia of Genes and Genomes; LC-MS, Liquid Chromatography–Mass Spectrometry; LiP-MS, Limited Proteolysis–Mass Spectrometry; LIPIDMAPs, LIPID Metabolites and Pathways Strategy; MALDI-MSI, Matrix-Assisted Laser Desorption/Ionization Mass Spectrometry Imaging; MAP4, MinHashed Atom-Pair fingerprint; MD, Molecular Dynamics; MHFP, MinHash Fingerprint; MiMeDB, Human Microbiome–Metabolome Database; MOFA, Multi-Omics Factor Analysis; MPI, metabolite–protein interaction; MS/MS, Tandem Mass Spectrometry; microbeMASST, Microbe Mass Spectrometry Search Tool; NMR, Nuclear Magnetic Resonance; NP3, NeuraIPLexer 3; PDB, Protein Data Bank; PredCMB, Predicting Changes in Microbial Metabolites; PROMIS, PROtein–Metabolite Interactions using Size separation; PUL, Polysaccharide Utilization Locus; QM/MM, Quantum Mechanics/Molecular Mechanics; QSAR, Quantitative Structure–Activity Relationship(s); SABIO-RK, System for the Analysis of Biochemical Pathways—Reaction Kinetics; SLC, solute carrier; SMILES, Simplified Molecular Input Line Entry System; SPR, Surface Plasmon Resonance; STITCH, Search Tool for Interacting Chemicals; TCA, tricarboxylic acid; TCDB, Transporter Classification Database; TPP, Thermal Proteome Profiling; TTD, Therapeutic Target Database

## Data Availability

No new datasets were generated for this review. The compound data for Fig. 2 were obtained from publicly available resources: MiMeDB and DrugBank. The processed compound lists and the scripts used to standardize structures and generate the density plots (RDKit 2025.03.3, seaborn 0.13.2) are available at: https://github.com/lejlagul/mpi-metabolite-drug-profiles
